# Norovirus-Associated Gastroenteritis Vesikari Score and Pre-Existing Salivary IgA in Young Children from Rural South Africa

**DOI:** 10.3390/v15112185

**Published:** 2023-10-30

**Authors:** Jean-Pierre Kabue, Ronewa Khumela, Emma Meader, Marcia Terezinha Baroni de Moraes, Afsatou Ndama Traore, Natasha Potgieter

**Affiliations:** 1 Department of Biochemistry and Microbiology, Faculty of Science, Engineering and Agriculture, University of Venda, Private Bag X5050, Thohoyandou 0950, South Africa; ronewakhumela@gmail.com (R.K.); afsatou.traore@univen.ac.za (A.N.T.); natasha.potgieter@univen.ac.za (N.P.); 2 Clinical Microbiology, Pathology Department, East Kent Hospitals University NHS Foundation Trust, Ashford TN24 OLZ, UK; emma.meader@nhs.net; 3 Laboratory of Comparative and Environmental Virology, Oswaldo Cruz Institute, Oswaldo Cruz Foundation (FIOCRUZ), Avenida Brazil, 4365-Manguinhos, Rio de Janeiro 21040-360, RJ, Brazil; marciaterezinha4@gmail.com

**Keywords:** vesikari score, specific IgA, diarrhea, NoV infections, symptomatic, asymptomatic, young children

## Abstract

Norovirus (NoV) is the leading cause of viral gastroenteritis, mostly affecting young children worldwide. However, limited data are available to determine the severity of norovirus-associated AGE (acute gastroenteritis) and to correlate it with the NoV-specific IgA antibodies’ level. Between October 2019 and September 2021, two hundred stool samples were randomly collected from symptomatic cases for the vesikari score and NoV-specific IgA assessment in young children from rural South Africa. Additionally, one hundred saliva specimens were concomitantly sampled within the same cohort to evaluate the NoV-specific salivary IgA levels. In addition, 50 paired saliva and stool samples were simultaneously collected from asymptomatic children to serve as controls. NoV strains in stool samples were detected using real-time RT-PCR, amplified, and genotyped with RT-PCR and Sanger sequencing. ELISA using NoV VLP (virus-like particles) GII.4 as antigens was performed on the saliva specimens. Dehydrated children were predominantly those with NoV infections (65/74, 88%; *p* < 0.0001). NoV-positive infections were significantly associated with the severe diarrhea cases having a high vesikari score (55%, 33/60) when compared to the non-severe diarrheal score (29.3%, 41/140; *p* < 0.0308). NoV of the GII genogroup was mainly detected in severe diarrhea cases (50.9%, 30/59; *p* = 0.0036). The geometric means of the NoV-specific IgA level were higher in the asymptomatic NoV-infected group (0.286) as compared to the symptomatic group (0.174). This finding suggests that mucosal immunity may not protect the children from the NoV infection. However, the findings indicated the contribution of the pre-existing NoV-specific IgA immune response in reducing the severity of diarrheal disease. A high vesikari score of AGE associated with the NoV GII genogroup circulating in the study area underscores the need for an appropriate treatment of AGE based on the severity level of NoV-associated clinical symptoms in young children.

## 1. Introduction

Diarrheal disease in children under 5 years of age is a public health concern in low-resource settings [1,2]. With the substantial decrease in rotavirus-associated diarrhea in the countries that have implemented routine rotavirus vaccination, NoV has emerged as the leading cause of AGE [3,4,5]. In South Africa, the rotavirus vaccination was introduced in 2009. 

NoV infections are responsible for approximately 700 million episodes of diarrhea annually worldwide with considerable morbidity and mortality. There is no specific treatment against NoV infections, and no vaccines are available [6,7]. Appropriate treatment of AGE based on the level of clinical symptoms associated with detected NoV infections [8] can help to avoid the complications of AGE and hospitalizations. Usually, in clinical practice, there is no confirmation of a viral AGE-causing agent in real-time, particularly in rural areas with limited laboratory facilities. The vesikari clinical severity system has been considered as the best predictor tool to categorize the severity of AGE and help to determine the treatment level of diarrheal disease [9,10,11]. However, data that include the severity scores for NoV-associated AGE in South Africa are scarce [12].

There are no licensed vaccines against NoV. Previous reports have demonstrated the evidence of vaccine-induced protective immunity against NoV in young children [13,14]. As NoV is an enteric virus that infects the mucosal surfaces of the gastrointestinal tract, it is evident that mucosal immunity will play a critical role in protection against NoV infection [15]. An effective enteric viral vaccine should induce a sufficient level of intestinal IgA antibodies to protect against viral infection in the gastrointestinal tract [16,17,18,19]. Previous NoV studies have demonstrated that the level of pre-existing salivary IgA antibodies correlated with minor risk of infection and restricted severity of gastroenteritis [18,20,21,22,23]. NoV investigations that evaluate the impact of pre-existing mucosal immunity in NoV infections are needed to contribute to the development of an effective NoV vaccine [15,22,23]. Virus-like particle (VLP)-derived vaccines are currently under development [21,22,23]. In South Africa, no data have been reported on the role of mucosal immunity, specifically IgA antibodies, in NoV infections among young children. Previous studies from rural communities in South Africa reported a high prevalence rate and diversity of Norovirus circulating associated with AGE [24,25]. In the present study, an Elisa-based method to detect NoV-specific IgA in the saliva samples was performed. The main finding was that NoV-specific mucosal levels correlated with limited severity of AGE.

This study aimed to determine the presence of pre-existing NoV-specific salivary IgA antibodies and corresponding NoV viral load and AGE vesikari score among young children from rural South Africa. 

## 2. Materials and Methods

### 2.1. Ethical Statement

The study protocols and consent procedures were approved by the ethics committees of the department of health in the Limpopo province, South Africa (Ref. LP_2018_07_016), and university of Venda (SMNS/19/MBY/05; SMNS/19/MBY/03). Written, informed consent of each participant was obtained from the parent or child guardian before stool and saliva samples were collected. An assent from each child was obtained in addition to informed consent from the parents/guardian. 

### 2.2. Study Design 

A cross-sectional study was performed on children under five years of age with or without diarrhea at different clinics and hospitals located within the rural areas of the Vhembe district in Limpopo, South Africa. A total of 30 primary health care clinics and 4 hospitals (Tshilidzini, Elim, Siloam, and Donald Fraser hospitals) were randomly designated sampling sites for this study. Bloody diarrhea cases were excluded from this study. Diarrhea was defined as 3 or more episodes of watery stool in the previous 24 h [8].

### 2.3. Sample Collection and Storage

From October 2019 to September 2021, two hundred (200) stool samples were collected from children under 5 years of age, with diarrhea, and then transported to the laboratory and stored at −20 °C until processing. Among these AGE cases, one hundred saliva specimens were concurrently sampled to evaluate the NoV-specific salivary IgA levels. In addition, fifty (50) saliva and (50) stool specimens were simultaneously collected from five-year-old children without diarrhea or healthy controls presenting at the clinics for immunization with no symptoms of AGE within 30 days before the enrolment. One of the study inclusion criteria was that the babies were not breastfed for at least 1 h before the saliva sampling. Samples from inpatient cases (hospitalized) were collected only from children admitted within 24 h to avoid nosocomial infection of norovirus. The study samples were randomly collected by the trained research assistants and qualified nurses. Cotton swabs were used to collect saliva and epithelial cells, by rubbing them inside of the cheek for around 1 min, from each study participant [26].

Prior to the ELISA assay, the saliva and epithelial cells were collected from the swabs by adding 1.5 mL of phosphate-buffered saline (PBS) (Thermo Scientific ^TM^ Oxoid^TM^, Basingstoke Hampshire, England), pH 7.2, to each tube containing the saliva specimen, followed by a vigorous 2800 rpm on the vortex (Vortex-5, Kylin-Bell lab instruments Co., Haimen, Jiangsu, China); then, the suspension of 1 mL was transferred to a new tube and kept at −20 °C as previously described [27]. Demographic data, clinical symptoms, and breastfeeding status of the participants were collected on pre-printed information forms.

### 2.4. RNA Extraction, NoV Detection, and Genotyping

The RNA extraction procedure was performed on all the stool specimens using the Boom method [28] prior to NoV detection and genotyping as previously described [24,25]. The RNA extracts were then subjected to the testing process for NoV detection with real-time PCR using a Corbet Research Rotor Gene 6000 platform [29]. To confirm the detected NoV strains, the genotyping procedures were performed as previously reported by Khumela et al. (2023) [29]. The PCR products of the amplified fragments were directly purified with a master mix of EXoSAP (Nucleics, Woollahra, NWS, Australia). All the purified amplicons were sent for partial sequencing at Ingaba BiotecTM (Pretoria, South Africa). The Sanger sequencing was performed using the same specific primers for the amplification on the ABI 3500XL Genetic Analyzer POP7^TM^ (Thermo-Scientific). The nucleotide sequences obtained were compared with the reference strains in the NCBI Genbank using the BLAST tool available at http://www.ncbi.nlm.nih.gov/blast (accessed on 13 March 2023), then analyzed for genotyping using Noronet typing tools (https://www.rivm.nl/mpf/typingtool/norovirus/) accessed on 15 March 2023 [30]. All the nucleotide sequences of NoV strains determined are available in the GenBank database under the accession numbers OQ048857.1-OQ048862.1; ON008179.1; OP600465.1-OP600467.1; OP257195.1; OM948744.1-OM948745.1; OM961396.1; OM961398.1-OM961399.1; OM970798.1-OM970799.1; OM970802.1; OM985015.1-OM985016.1; and OM993270.1-OM993271.1.

### 2.5. Vesikari Scoring System for Assessment of Severity of NoV-Associated AGE

To evaluate the severity of NoV-associated AGE, we used the vesikari score system (Table 1) as previously described [9,10]. The following clinical data were recorded to calculate the score: the number and duration of diarrhea and vomiting episodes, the maximum body temperature, severity of dehydration, and treatment modalities. The classification of dehydration severity was carried out by the qualified nurses on the basis of the loss of body weight (1–5%: moderate dehydration; ≥6%: severe dehydration) and the dehydration treatment based on the dehydration level (mild dehydration: treat at home by giving liquid and food; moderate dehydration: treat using ORS solution in the clinic; severe dehydration: treat using IV therapy administered at the clinic) [10,31,32]. Any score below seven was considered as mild acute gastroenteritis, scores between seven and ten were classified as moderate acute gastroenteritis, and scores equal to or higher than eleven were categorized as severe acute gastroenteritis.

### 2.6. Determination of NoV-Specific Salivary IgA Antibodies

Salivary IgA antibodies against NoV antigens were measured with an enzyme-linked immunosorbent assay (ELISA) as previously reported [33]. The level of pre-existing anti-NoV antibodies in saliva samples was determined using NoV VLP GII.4 as antigens (cat. REC31620-100, The Native Antigen Company, Kidlington, Oxfordshire, UK). The saliva IgA measurement was evaluated in duplicate wells for each sample. Briefly, 100 µL of NoV VLPs at 2 µg/mL in a carbonate/bicarbonate buffer (pH 9.6) was plated and incubated at 4 °C overnight. On the following day, the plates were washed three times with PBS-T (PBS containing 0.05% of Tween 20) and blocked for 1 h at 37 °C with PBS-T 3% FBS (fetal bovine serum), then incubated with serial dilutions (from 1/20 to 1/60) of saliva samples (100 µL per well) in PBS-T 1% FBS for 1.5 h at 37 °C and with NoV-specific IgA anti-human antibodies conjugated with Horseradish peroxidase (HRP) (Sigma-Aldrich, St.Louis, MI, USA) at a dilution of 1/4000 in PBS-T 1% FBS for 1 h at 37 °C. Boiled saliva samples a were used as negative controls. The last washing step in four times was performed with PBS-T. The detection reaction of the bound antibody was revealed with the addition of 50 µL of o-phenylenediamine (Sigma) and stopped at 10 min with 3 M H_2_SO_4_ (Murula Services, Johannesburg, South Africa). The absorbance was read directly within 5 min at 492 nm using a microplate reader, EMax^R^Plus (Molecular devices, San Jose, CA, USA, ISO 9001).

### 2.7. Statistical Analysis

The geometric means’ level of NoV-specific salivary IgA in infected individuals and the Pearson correlation coefficients to evaluate the correlation between the different groups were calculated using Excel software 365 and GraphPad prism 9 (GraphPad Inc., San Diego, CA, USA). The viral load CT values were determined using Rotor-gene Q-Rex software 2.3.1 to assess the difference between the symptomatic and asymptomatic groups. Using Chi-square and Fisher’s exact tests, the *p*-values were calculated.

## 3. Results

### 3.1. Study Characteristics

During the study period, 200 stool samples were collected from young children with diarrhea. The demographic characteristics and clinical features associated with AGE are presented in Table 2. The median age of the study population was 10 months (range: 1–41 months) and the gender distribution was 58% (116/200) male and 42% (84/200) female. Most children positive for norovirus had at least three episodes of diarrhea per day (73%, 54/74; *p* = 0.1541) and 2–4 episodes of vomiting within 24 h (49%, 36/74; *p* = 0.742). The majority of samples (161/200, 80.5%) with diarrhea were collected at the date of onset between 1 and 3 days (interval between the onset of diarrhea and the stool collection date), which is known as the peak period of viral shedding. Most norovirus-positive children had a body temperature below 37 °C (95%, 70/74) on admission to the health care center. Children with signs of dehydration were predominantly affected (88%, 65/74; *p* < 0.0001) by NoV infection.

**Figure 1:**
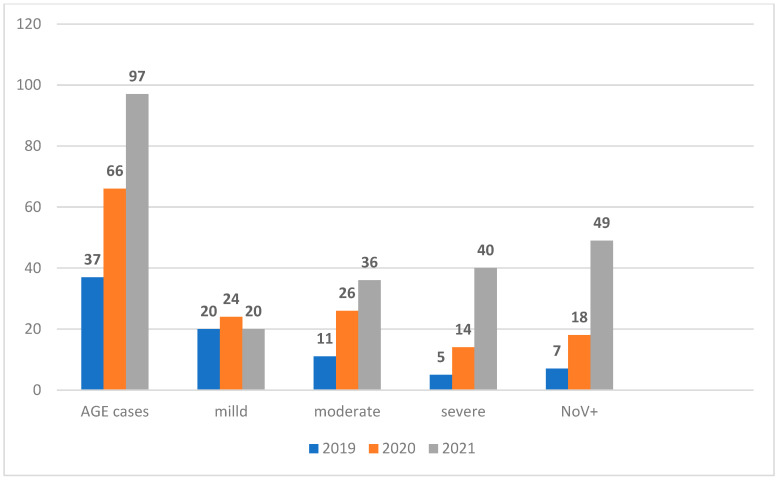
Distribution of vesikari severity score based on the year of AGE onset between 2019 and 2021.

There was an increase in NoV-positive dehydrating diarrhea cases over the years with 7/37 (18.9%) cases in 2019, 18/66 (27.3%) cases in 2020, and 49/97 (50.5%) cases in 2021 (Figure 1). Hospitalized patients were frequently infected with NoV (59%, 44/74) when compared to the outpatients (41%, 30/74; *p* = 0.0187) (Table 2). Furthermore, children with severe vesikari scores of AGE cases were mostly hospitalized (44.8%, 43/96) as compared to outpatients (16.3%, 17/104).

In this study, the assessment of severity of NoV-associated AGE showed that NoV-positive diarrhea cases were significantly associated with the severe vesikari score (55%, 33/60) when compared to the non-severe diarrheal score (29.3%, 41/140; *p* < 0.0308) (Table 3).

In addition, the evaluation of different NoV genogroup distributions among diarrheal severity levels revealed that NoV genogroup II was predominantly associated with severe diarrhea cases (50.9%, 30/59; *p* = 0.0036), whereas NoV genogroup I was mostly found regarding diarrhea with a mild clinical severity score (66.7%, 6/9; *p* = 0.0036) (Table 4).

### 3.2. NoV Genogroups and Genotypes in Young Children Recruited for IgA Assessment

The predominance of NoV genogroup II (37/100; 37%) comprising GII.4. Sydney 2012 capsid genotypes was observed among young children. The GI genogroup (4/100; 4%) and GII/GI mixed genogroup (3/100; 3%) were also found (Table 5). Only one capsid genotype, GII.4 Sydney 2012, was detected and successfully genotyped. In this study, PCR inhibition that could have influenced Ct values was monitored with the use of an internal control and all the control Ct values were within the 33–34 cycle range.

### 3.3. NoV GII.4 Specific Salivary IgA in Young Children

**Figure 2:**
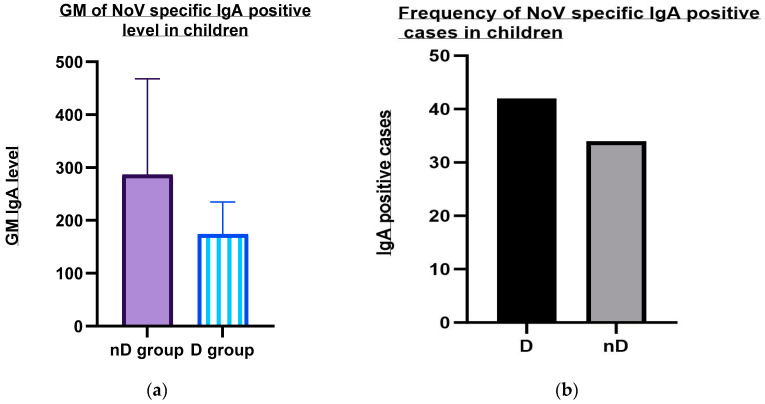
(**a**) Comparison of pre-existing NoV-specific salivary IgA level in young children with or without diarrhea. GM, Geometric mean (adjusted scale: GM X 1000); D, Diarrhea; and nD, Non-diarrhea. (**b**). Comparison of pre-existing NoV-specific salivary IgA frequency in young children with or without diarrhea. D, Diarrhea; nD, Non-diarrhea.

The comparison of the percentage of positivity rates and titers of NoV-specific IgA among the groups revealed the following:

The geometric means of the NoV-specific IgA level were higher in the asymptomatic NoV-infected group (0.286) as compared to the symptomatic group (0.174) (Figure 2a).

There was no significant difference of NoV infection with IgA-positive cases between the symptomatic (34/100; 34%) and asymptomatic group (21/50; 42%) (Figure 2b). Also, the difference between the number of breastfed children who were IgA-positive (21/100; 21%) and IgA-positive cases of children not breastfed (14/100; 14%) was not significant.

## 4. Discussion

NoV is now recognized as the leading cause of AGE worldwide [34,35,36]. However, limited data are available to determine the vesikari severity score of AGE associated with NoV infection and the level of pre-existing NoV-specific IgA.

Aiming to assess the pre-existing NoV-specific IgA and the AGE vesikari score in young children, this study demonstrated that the NoV-positive severe diarrhea cases were significantly associated with a high vesikari score (55%, 33/60) when compared to the non-severe norovirus-positive diarrhea cases (29.3%, 41/140; *p* < 0.0308; Table 3). As expected, children with severe NoV-associated AGE were predominantly hospitalized patients (59%, 44/74) when compared to the outpatients (41%, 30/74; *p* = 0.0187; Table 2). In addition, NoV GII was the predominant genotype found to be associated with severe diarrhea cases (50.9%, 30/59) while NoV GI was mostly recovered regarding diarrhea with a mild clinical severity score (66.7%, 6/9; *p* = 0.0036; Table 4). This finding agrees with previous NoV investigations, which reported a significant difference of the estimated GII (not GI) viral load between symptomatic and asymptomatic groups of patients, suggesting the involvement of GII strains in AGE [12,30,37,38,39]. The findings are in accordance with previous studies that reported GII as the worldwide predominant genogroup involved in severe clinical cases [39,40].

Since 2019 to 2021, the decline in NoV infections has been reported in several countries [41,42,43,44,45,46] with the implementation of COVID-19 public health interventions to mitigate the pandemic. However, the current study revealed the enhanced NoV activity in 2021 as well as the increased severity score of AGE cases from 13.5% in 2019 to 50.5% in 2021 (Figure 1). Similarly, Chan (2022) demonstrated the return of norovirus and rotavirus episodes in winter 2020–2021 in Hong Kong while multiple non-pharmaceutical interventions for COVID-19 were still in effect [47]. Factors associated with the possible emergence of new immune-escaped strains and host–virus interactions [48,49] may be considered.

NoV epidemiology is evolving due to the genetic diversity, the continuous mutation changes, and genome recombination of the circulating strains [50,51,52,53,54]. Though previous studies have reported NoV causing acute gastroenteritis associated with moderate to severe diarrhea disease [55,56], the current study, compared to other recent AGE etiology investigations, found NoV infections to be associated with severe AGE [57,58,59]. Of note, previous NoV surveys in the study area did not report a high presence of dehydrating diarrhea as revealed in the current study [25,60]. There was a rise in dehydration cases associated with an enhanced NoV activity over years in the study area (Figure 1). Furthermore, most NoV-infected children had 2–4 episodes of vomiting within 24 h (49%, 36/74; *p* = 0.742). This finding is consistent with a recent study from Argentina, which found that watery diarrhea, complete vaccination against rotavirus, and vomiting were three key parameters mostly associated with possible NoV gastroenteritis [61]. Dehydration is the most severe threat and the major complication of AGE [62]. The presence of dehydration in AGE reflects the severity of diarrhea disease as previously reported [63] and requires aggressive management of NoV-associated AGE [64]. WHO recommends the degree of therapy for AGE based on its severity of dehydration [65].

IgA is one of the first lines of defense that help to stop infective pathogens from invading the mucosal barrier [66,67]. Salivary IgA has been previously shown to be reflective of NoV mucosal immunity [20,68,69,70]. However, there is a lack of data on the pre-existing specific salivary IgA titers in young children and their impact in the NoV-associated AGE. In this survey, only the VLP of the worldwide circulating and predominant genotype GII.4 [71,72] was used as an antigen for ELISA assays though the ongoing vaccine trials that are using GII.4c and GI.1 genotypes [73]. Children aged less than 12 months in our sample collection as previously described were not excluded in this study [26,74]. Several conflicting findings have been reported on the effect of breastfeeding in childhood NoV-associated AGE. Recently, Vielot et al. [75] found that exclusive breastfeeding was rare and could not prevent norovirus or Sapovirus AGE in a Nicaraguan birth cohort, which contradicted several previous studies [76,77,78].

In this study, the geometric means’ level of NoV GII.4-specific salivary IgA was higher in the asymptomatic NoV-infected group when compared to the symptomatic group. Ramani and coworkers [16] reported similar findings, although their investigation was based solely on healthy adult human volunteers. These findings suggest that the pre-existing NoV GII.4-specific IgA level is associated with reduced symptoms or severity of gastroenteritis. The study results are in accordance with the previous NoV-specific IgA investigations reported in USA and Peru, which demonstrated that the pre-existing IgA does not protect individuals from NoV infection but may limit viral replication [20,79]. Costantini and colleagues [68] reported an increase in NoV-specific IgA titers with a similar pattern in both symptomatic and asymptomatic participants at day 5 after onset during outbreaks. They did not observe the correlation between viral load, disease severity, and immune response. Their findings could be related to the age group of the participants (adults), settings (long-term care facilities where the exposure is expected to be more common), and the context of outbreaks.

This study has some limitations including the small sample size that could not allow us to comprehensively analyze different variables listed in the study participant characteristics as well as the lack of the longitudinal investigation that could help to monitor the variations of the IgA level and vesikari scores over time throughout the NoV infection as previously reported [69,70]. Furthermore, the random sampling method used in this survey could not help us to differentiate the sporadic cases from NoV outbreaks throughout the study period. We did not screen other enteric pathogens, which could be associated as coinfections with AGE in the study area [80].

To our knowledge, this survey is the first cross-sectional study on the pre-existing NoV-specific salivary IgA in asymptomatic and symptomatic young children. Previous reports focused on adult volunteers and mostly on outbreak cases. Despite the small number of individuals tested for salivary NoV-specific IgA, the study results support the evidence that NoV mucosal immunity is associated with reduced severity of symptoms. The extent to which the vaccine inducing NoV IgA alone protects against infection remains to be determined [81]. The findings from this study inform possible preventive strategies against NoV-related AGE in young children and encourage the need for vaccine adjuvants [82] that may promote mucosal IgA response [6] for an effective NoV vaccine. The high vesikari severity score of AGE was predominantly associated with the NoV GII genogroup in this study. More investigations are needed to confirm the severity of AGE associated with NoV infection and accordingly adjust the therapy as well as the preventive measures against diarrhea.

## Figures and Tables

**Table 1:** Vesikari clinical severity scoring system [9,11].

Parameters	0	1	2	3
Diarrhea				
-Maximum frequency number of stools per day	0	1–3	4–5	≥6
-Diarrhea duration (day)	0	1–4	5	≥6
Vomiting				
-Maximum number per day	0	1	2–4	≥5
-Vomiting duration (day)	0	1	2	≥3
Maximum body temperature (°C)	≤37	37.1–38.4	38.5–38.9	≥39.0
Severity of dehydration (%)	N/A	N/A	1–5	≥6
Treatment	None	Rehydration	Hospitalization	N/A
Severity rating scales		<7 (mild)	7–10 (moderate)	≥11 (severe)

**Table 2:** Clinical features of AGE in young children from Vhembe district, South Africa.

Variables	Total (%), *n* = 200	Norovirus-Positive, *n* = 74 (37%)	Norovirus-Negative, *n* = 126 (63%)	*p*-Value
Diarrhea				
-Maximum frequency number of stools per day				
3	133	54 (73)	79 (63)	*p* = 0.1541
3–5	46	16 (22)	30 (24)	
≥6	21	4 (5)	17 (13)	
Diarrhea duration (days)				
1–4	182	66 (89)	114 (90)	*p* = 0.8096
≥5	18	8 (11)	12 (10)	
Interval (days)				
1–3	161 (81)	61 (82)	100 (79)	*p* = 0.7123
>3	39 (20)	13 (18)	26 (21)	
Vomiting				
-Maximum number per day				
0	81	23 (31)	58 (46)	*p* = 0.742
1	30	15 (20)	15 (12)	
≥3	89	36 (49)	53 (42)	
-Vomiting duration				
1	38	18 (24)	20 (16)	*p* = 0.7925
2	37	15 (20)	22 (17)	
≥3	44	18 (24)	26 (21)	
Maximum body temperature				
≤37	170	70 (95)	100 (79)	*p* = 0.0036 *
≥37	30	4 (5)	26 (21)	
Dehydration (as assessed by the nurses)				
No dehydration	71	9 (12)	62 (49)	*p* < 0.0001 *
Dehydration	129	65 (88)	64 (51)	
Non-severe	66	29 (39)	37 (29)	*p* = 0.1601
Severe	63	36 (49)	27 (21)	
Treatment				
None	62	17 (23)	45 (36)	*p* = 0.0551
ORS	79	32 (43)	47 (37)	
IVF	30	9 (12)	21 (17)	
IVF/ORS	29	16 (22)	13 (10)	
Setting				
Hospitalized	96 (48)	44 (59)	52 (41)	*p* = 0.0187 *
Outpatients	104 (52)	30 (41)	74 (59)	

* *p*-Value is significant.

**Table 3:** Association between vesikari score and NoV infections.

AGE Severity (Ruuska Score)	Diarrhea Case Number (n = 200)	Norovirus-Positive (%)	*p*-Values
Mild (<7)	65	17 (26)	*p* = 0.5976
Moderate (7–10)	75	24 (32)	
Mild (<7)	65	17 (26)	*p* = 0.0437 *
Severe (≥11)	60	33 (55)	
Moderate (7–10)	75	24 (32)	*p* = 0.1138
Severe (≥11)	60	33 (55)	
Non-Severe (<11)	140	41 (29)	*p* < 0.0308 *
Severe (≥11)	60	33 (55)	

* *p*-Value is significant.

**Table 4:** Distribution of diarrheal severity by NoV genogroups and genotypes.

Severity Score				Total (%)
	Mild (%)	Moderate (%)	Severe (%)	
NoV Genogroup	17 (23)	24 (32)	34 (45)	74 (100)
GI	6 (67)	2 (22)	1 (11)	9
GII	9 (15)	20 (34)	30 (51)	59
GI/GII	2 (33)	2 (33)	2 (33)	6
NoV Genotypes				
GII.4 Sydney 2012	6 (32)	6 (32)	7 (37)	19

**Table 5:** Distribution of NoV genogroups and genotypes among the participants recruited for IgA assessment.

	Symptomatic (n = 100)	Asymptomatic (n = 50)
	NoV-Positive (%)	NoV-Positive (%)
NoV Genogroup	**44 (44)**	**10 (20)**
GI	4 (4)	4 (8)
GII	37 (37)	6 (12)
GI/GII	3 (3)	0 (0)
NoV Genotypes		
GII.4 Sydney 2012	21 (45.7)	2 (16.7)

## Data Availability

The data set considered during this survey is available upon request to the corresponding author.
